# Supplementary material for: Ciprofloxacin and Trimethoprim Adsorption/Desorption in Agricultural Soils
Source: Int J Environ Res Public Health. 2022 Jul 10;19(14):8426. doi: 10.3390/ijerph19148426 (PMC9318069; doi:10.3390/ijerph19148426)
Supplement: Supplementary file 1 [file ijerph-19-08426-s001.zip › ijerph-1799181-supplementary.pdf]

Supplementary Material

Ciprofloxacin and Trimethoprim adsorption/desorption in agricultural soils

Lucía Rodríguez-López[1]*, Vanesa Santás-Miguel[1], Raquel Cela-Dablanca[2], Avelino Núñez-Delgado[2], Esperanza Álvarez-Rodríguez[2], Paula Pérez-Rodríguez[1], Manuel Arias-Estévez[1]

[1]Soil Science and Agricultural Chemistry, Faculty of Sciences, University of Vigo, 32004 Ourense, Spain
[2]Deptartment of Soil Science and Agricultural Chemistry, Engineering Polytechnic School, University of Santiago de Compostela, 27002 Lugo, Spain

*Correspondence: Lucía Rodríguez-López (email: lucia.rodriguez.lopez@uvigo.es)

Conflict of interest: none

Table S1. Ciprofloxacin (CIP) adsorbed in the equilibrium, expressed in µmol kg^{-1} (and in percentage, into brackets) for each of the initial concentrations added (C_0) and for each of the 17 soils studied. Mean, median, maximum and minimum values are referred to adsorption percentages.

	**C_0 (µmol L^{-1})**						
**Soil**	**5**	**10**	**25**	**50**	**100**	**200**	**400**
1	477.2 (96.5)	808.6 (97.1)	1803.5 (97.2)	3620.0 (96.0)	7059.4 (93.6)	13,611.6 (80.1)	16,946.8 (56.3)
2	482.5 (95.8)	788.7 (97.0)	1911.9 (97.7)	3727.9 (97.8)	8526.2 (96.3)	15,252.0 (93.1)	22,276.0 (79.1)
3	455.5 (96.4)	767.5 (97.2)	1819.7 (97.4)	3688.5 (95.9)	7099.6 (94.3)	13,749.4 (90.1)	25,014.8 (75.1)
4	433.5 (96.2)	844.3 (96.6)	1971.4 (96.3)	3868.6 (93.6)	7193.5 (91.3)	13,133.3 (85.9)	22,288.6 (72.6)
5	469.5 (96.9)	763.9 (97.7)	1826.9 (97.7)	3780.6 (97.2)	6876.5 (96.0)	14,472.9 (93.7)	26,012.2 (84.6)
6	470.1 (96.7)	764.7 (97.6)	1855.5 (98.0)	3126.6 (97.9)	7098.2 (95.6)	13,929.4 (92.1)	24,221.8 (80.8)
7	471.3 (96.4)	802.9 (96.8)	1946.2 (95.2)	3579.7 (94.9)	7437.8 (91.5)	13,762.9 (86.3)	23,217.0 (79.4)
8	435.6 (95.8)	838.6 (97.4)	1792.6 (97.9)	3478.1 (97.1)	8459.4 (95.9)	14,135.2 (93.0)	25,479.3 (82.9)
9	355.2 (94.7)	895.7 (97.2)	2439.6 (98.0)	5016.1 (97.5)	7799.8 (96.4)	13,842.6 (88.2)	26,170.9 (80.0)
10	339.6 (97.2)	859.0 (94.6)	2488.8 (96.0)	4708.4 (96.0)	7652.3 (93.9)	13,004.6 (83.8)	24,309.6 (77.5)
11	441.5 (96.8)	783.8 (98.0)	1952.6 (98.2)	3590.9 (98.4)	8367.9 (96.9)	14,282.1 (93.2)	26,221.9 (84.4)
12	355.4 (96.6)	882.7 (98.0)	2364.0 (98.7)	4701.4 (98.9)	7376.9 (97.7)	14,439.5 (94.7)	26,012.3 (86.7)
13	370.0 (95.6)	832.4 (96.4)	2306.8 (98.0)	5556.7 (98.0)	7491.6 (97.3)	14,375.4 (93.6)	26,590.1 (84.4)
14	426.5 (92.2)	754.5 (92.7)	1682.6 (86.0)	3288.8 (85.4)	6652.6 (83.0)	12,330.6 (76.8)	20,796.0 (67.8)
15	432.7 (96.4)	801.5 (97.5)	1890.3 (97.5)	3842.1 (96.8)	7476.7 (95.2)	14,607.8 (94.7)	27,103.8 (89.2)
16	455.7 (97.4)	819.1 (98.2)	1931.1 (98.6)	5132.5 (98.3)	7759.2 (97.8)	15,549.4 (96.9)	28,701.7 (94.5)
17	439.5 (100.0)	757.6 (95.8)	1818.3 (93.8)	3626.9 (90.5)	6247.6 (84.4)	12,821.8 (79.0)	22,546.3 (69.0)
Mean	96.3	96.8	96.6	95.9	93.9	89.1	79.1
Median	96.4	97.2	97.7	97.1	95.6	92.1	80.0
Mode	96.4	97.2	98.0	96.0	83.0	94.7	84.4
Minimum	92.2	92.7	86	85.4	83	76.8	56.3
Maximum	100	98.2	98.7	98.9	97.8	96.9	94.5

Table S2. Trimethoprim (TRI) adsorbed in the equilibrium, expressed in µmol kg^{-1} (and in percentage, into brackets) for each of the initial concentrations added (C_0) and for each of the 17 soils studied. Mean, median, maximum and minimum values are referred to adsorption percentages.

	C_0 (µmol L^{-1})						
**Soil**	**2.5**	**5**	**10**	**20**	**30**	**40**	**50**
1	55.7 (27.9)	92.1 (22.9)	149.2 (19.0)	271.1 (16.8)	318.0 (13.7)	430.2 (14.1)	528.6 (13.3)
2	59.0 (30.2)	104.6 (26.6)	194.8 (23.9)	327.2 (20.6)	446.9 (19.5)	581.1 (19.0)	625.6 (15.8)
3	64.8 (32.3)	119.8 (30.4)	227.8 (28.6)	419.9 (26.6)	513.9 (21.7)	705.7 (22.6)	893.8 (22.0)
4	60.2 (30.5)	105.4 (26.0)	188.1 (23.3)	341.8 (21.1)	450.7 (18.9)	588.0 (18.7)	758.0 (18.4)
5	81.9 (41.4)	145.3 (36.2)	248.5 (30.9)	482.7 (30.2)	672.8 (28.4)	790.0 (25.6)	1018.2 (25.6)
6	96.5 (49.7)	170.4 (42.0)	280.0 (35.5)	557.0 (34.2)	678.4 (29.5)	815.5 (26.4)	1037.2 (25.6)
7	73.1 (37.8)	132.8 (32.8)	229.0 (28.9)	428.5 (26.5)	555.9 (23.8)	691.2 (21.8)	802.3 (19.9)
8	83.6 (42.3)	146.9 (36.8)	256.1 (32.5)	444.9 (28.0)	635.1 (26.9)	796.4 (24.8)	997.9 (25.4)
9	83.4 (41.7)	147.5 (35.0)	249.2 (31.7)	416.7 (27.6)	581.5 (24.1)	704.7 (22.2)	820.4 (20.1)
10	62.2 (33.3)	146.1 (34.8)	190.9 (24.5)	331.8 (21.4)	487.5 (20.4)	623.4 (19.1)	674.6 (16.8)
11	59.1 (29.3)	103.7 (26.6)	186.9 (23.7)	324.2 (20.6)	453.0 (19.0)	569.2 (18.1)	705.7 (18.0)
12	68.6 (33.6)	128.4 (31.0)	205.2 (26.4)	371.5 (23.7)	564.3 (23.4)	702.3 (21.9)	772.5 (19.5)
13	63.6 (31.8)	111.1 (26.6)	172.6 (22.4)	311.5 (20.0)	483.0 (19.8)	566.1 (18.2)	641.4 (15.9)
14	91.3 (45.3)	111.2 (25.8)	155.8 (18.3)	236.7 (15.8)	321.5 (13.5)	503.6 (15.5)	575.1 (14.5)
15	125.0 (62.6)	174.1 (40.4)	355.0 (41.6)	468.2 (32.5)	778.9 (32.4)	916.9 (28.6)	998.2 (24.9)
16	151.2 (74.3)	208.7 (48.4)	416.4 (48.3)	643.8 (43.9)	1050.2 (43.3)	1265.4 (39.4)	1586.9 (39.6)
17	80.5 (39.6)	104.7 (24.5)	248.2 (29.4)	327.8 (22.5)	528.6 (21.8)	689.4 (20.8)	720.9 (18.0)
Mean	40.2	32.2	28.8	25.4	23.5	22.2	30.4
Median	37.8	31.0	28.6	23.7	21.8	21.8	19.9
Mode	27.9	26.6	18.3	20.6	13.5	14.1	25.6
Minimum	27.9	22.9	18.3	15.8	13.5	14.1	13.3
Maximum	74.3	48.4	48.3	43.9	43.3	39.4	39.6

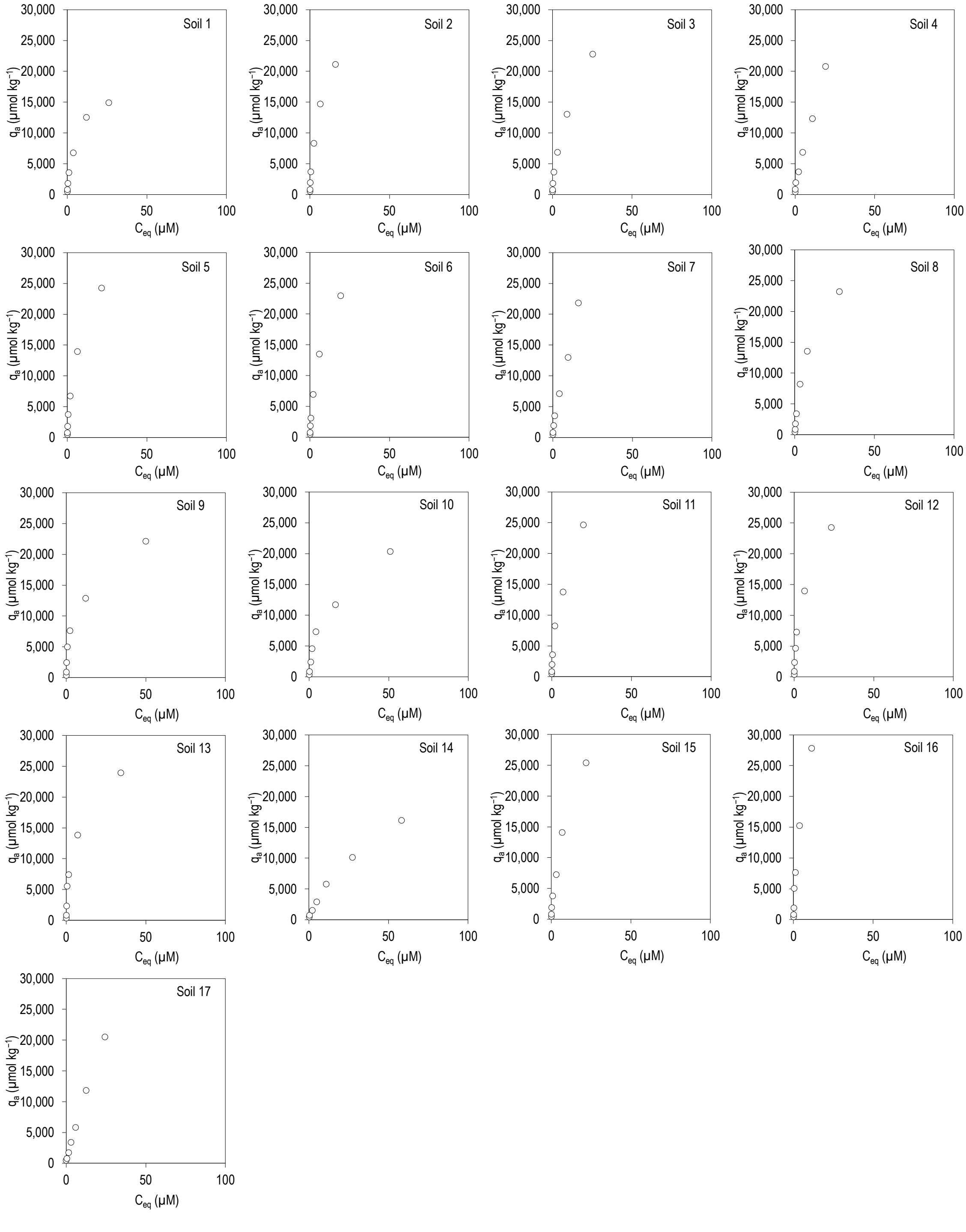

Figure S1. Desorption curves for Ciprofloxacin (CIP) in the 17 soils studied. q_a: CIP adsorbed onto the soil after a desorption cycle; C_{eq}: CIP concentration in the equilibrium solution.

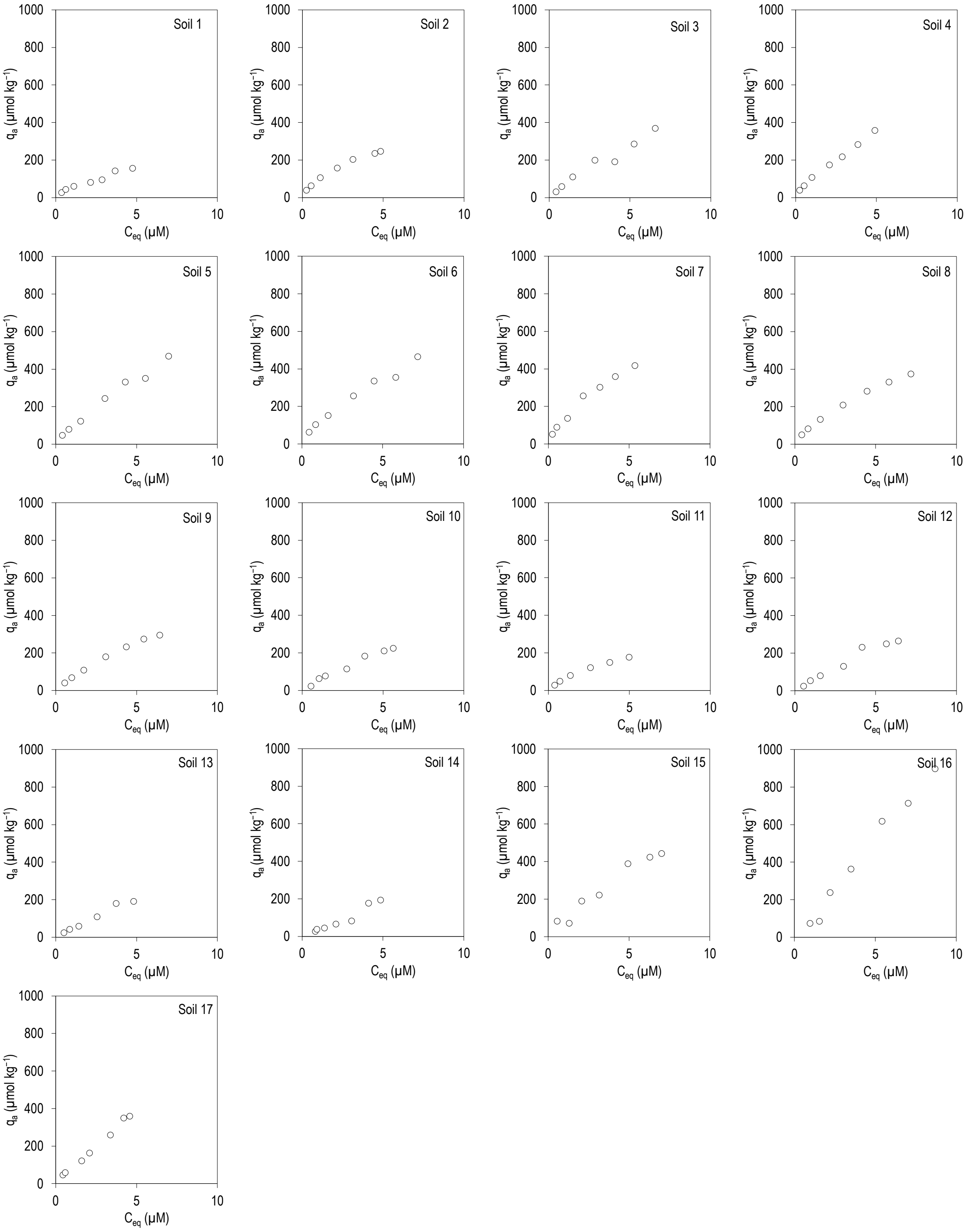

Figure S2. Desorption curves for Trimethoprim (TRI) in the 17 soils studied. q_a: TRI adsorbed onto the soil after a desorption cycle; C_{eq}: CIP concentration in the equilibrium solution.
